# Recent Insights and Recommendations for Preventing Excessive Gestational Weight Gain

**DOI:** 10.3390/jcm13051461

**Published:** 2024-03-02

**Authors:** Magdalena Niebrzydowska-Tatus, Aleksandra Pełech, Anna K. Rekowska, Małgorzata Satora, Angelika Masiarz, Zuzanna Kabała, Żaneta Kimber-Trojnar, Marcin Trojnar

**Affiliations:** 1 Department of Obstetrics and Perinatology, Medical University of Lublin, 20-090 Lublin, Poland; mniebrzydowska7@gmail.com (M.N.-T.); apilszyk@gmail.com (A.P.); 2 Student’s Scientific Association and Department of Obstetrics and Perinatology, Medical University of Lublin, 20-090 Lublin, Poland; arekowska@icloud.com (A.K.R.); msatoraa@gmail.com (M.S.); masiarz.ang@gmail.com (A.M.); zuzanna.kabala00@gmail.com (Z.K.); 3 Department of Internal Diseases, Medical University of Lublin, 20-059 Lublin, Poland; marcin.trojnar@umlub.pl

**Keywords:** excessive gestational weight gain, obesity, BMI, fetal programming

## Abstract

Recommendations for weight gain during pregnancy are based on pre-pregnancy body mass index (BMI). Pregnancy is a risk factor for excessive weight gain and many endocrine problems, making it difficult to return to pre-pregnancy weight and increasing the risk of postpartum obesity and, consequently, type 2 diabetes and metabolic syndrome. Both excessive gestational weight gain (EGWG) and obesity are associated with an increased risk of gestational hypertension, pre-eclampsia, gestational diabetes, cesarean section, shoulder dystocia, and neonatal macrosomia. In the long term, EGWG is associated with increased morbidity and mortality, particularly from diabetes, cardiovascular disorders, and some cancers. This study aims to present recommendations from various societies regarding weight gain during pregnancy, dietary guidance, and physical activity. In addition, we discuss the pathophysiology of this complication and the differential diagnosis in pregnant women with EGWG. According to our research, inadequate nutrition might contribute more significantly to the development of EGWG than insufficient physical activity levels in pregnant women. Telehealth systems seem to be a promising direction for future EGWG prevention by motivating women to exercise. Although the importance of adequate pre-pregnancy weight and weight gain during pregnancy is well known, an increasing number of women gain excessive weight during pregnancy.

## 1. Introduction

Nowadays, excessive gestational weight gain (EGWG) and obesity are one of the most important obstetric problems, increasing the risk of maternal as well as fetal complications. Obesity increases the risk of gestational diabetes mellitus (GDM), gestational hypertension, and thromboembolic disease. Newborns of diabetic mothers are more likely to have macrosomia associated with perinatal injuries such as shoulder dystocia. It is worth mentioning that as body mass index (BMI) increases, the number of preterm births and cesarean sections increases [1,2,3].

During pregnancy, weight gain physiologically occurs, regardless of the baseline value. Guided by the welfare of the developing fetus, a pregnant woman should not reduce her weight. Women are advised to plan pregnancies when they are in the best possible health. Those suffering from chronic diseases should be advised that pregnancy should occur during a period of remission or when symptoms are under control [4]. Obesity is classified as a chronic disease, so it should be recommended to normalize weight before pregnancy. An important aspect is that in many cases, pregnancy is a critical period, and if excessive weight gain occurs during it, this often becomes a cause of lifelong obesity. In the long term, it is associated with increased morbidity and mortality, especially from diabetes, cardiovascular disorders, and cancers.

As already mentioned, weight reduction during pregnancy is not recommended, although preventing excessive weight gain during this period can reduce the risk of obstetric complications. Recommendations for weight gain in pregnancy are based on pre-pregnancy BMI. Based on the American guidelines of the International Federation of Gynecology and Obstetrics (FIGO), the recommended weight gain for pregnant women with a previously normal BMI (18.5–25 kg/m^2^) is 11.5–16.0 kg. For overweight and obese women, it is recommended to be 7–11.5 kg and 5–9 kg, respectively (Table 1). In twin pregnancies, total weight gain and weekly weight gain in the second and third trimesters should be about 10% higher [5].

The purpose of our review was to discuss the underlying factors behind the occurrence of EGWG and its long-term health consequences, with an emphasis on research seeking the most effective strategies that can prevent EGWG.

## 2. Material and Methods

We conducted a comprehensive literature review using Pubmed database, considering articles written in English and Polish published between January 2013 and November 2023. However, if an article we found on PubMed provided an abstract that seemed to contain information relevant to the study topic and its full version was not linked, we tried to search for it via Google Scholar for better access. Inclusion criteria involved randomized clinical trials, meta-analyses, systematic reviews, human studies, and recommendations of scientific societies. Exclusion criteria: duplicated papers, case reports, conference abstracts, comments, articles not accessible as full-text, and language other than English and Polish.

We used the following keywords and their combinations: “excessive gestational weight gain”, “risk factors”, “psychosocial factors”, “pathophysiology”, “adipokines”, “insulin resistance”, “diabetes mellitus”, “obesity”, “overweight”, “PCOS”, “diet”, “nutrition”, “training”, “physical activity”, “recommendations”, “maternal consequences”, “long-term problems”,” postpartum”, “weight retention”, “metabolic syndrome”, “depression”, “fetal programming”, “fetal development”, “metabolic programming”, “pre-pregnancy”, “BMI”. After applying inclusion and exclusion criteria, we selected 114 research works for analysis.

## 3. Results

**Figure 1:**
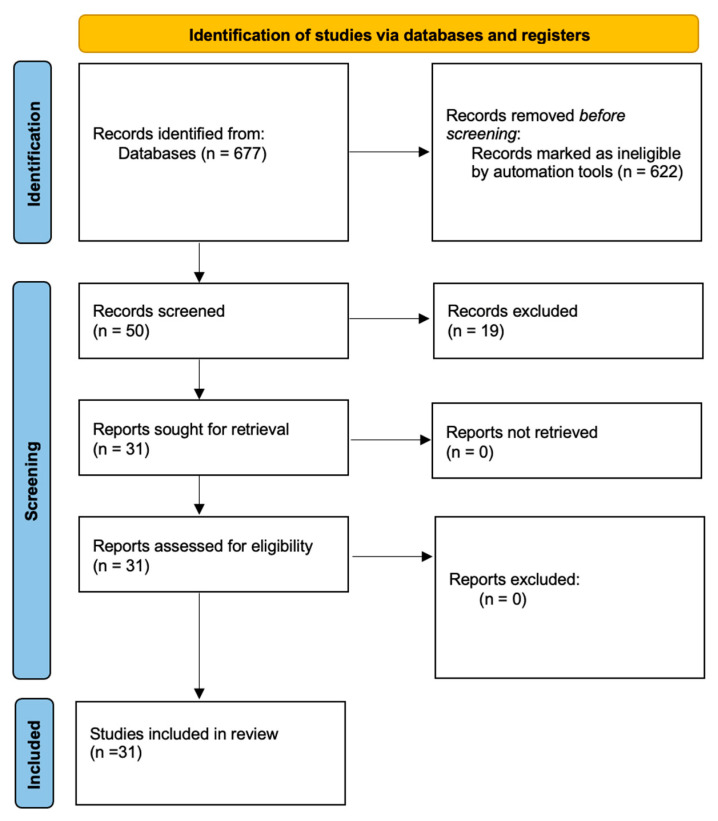
Study selection for EGWG prevention—dietary and training recommendations.

**Figure 2:**
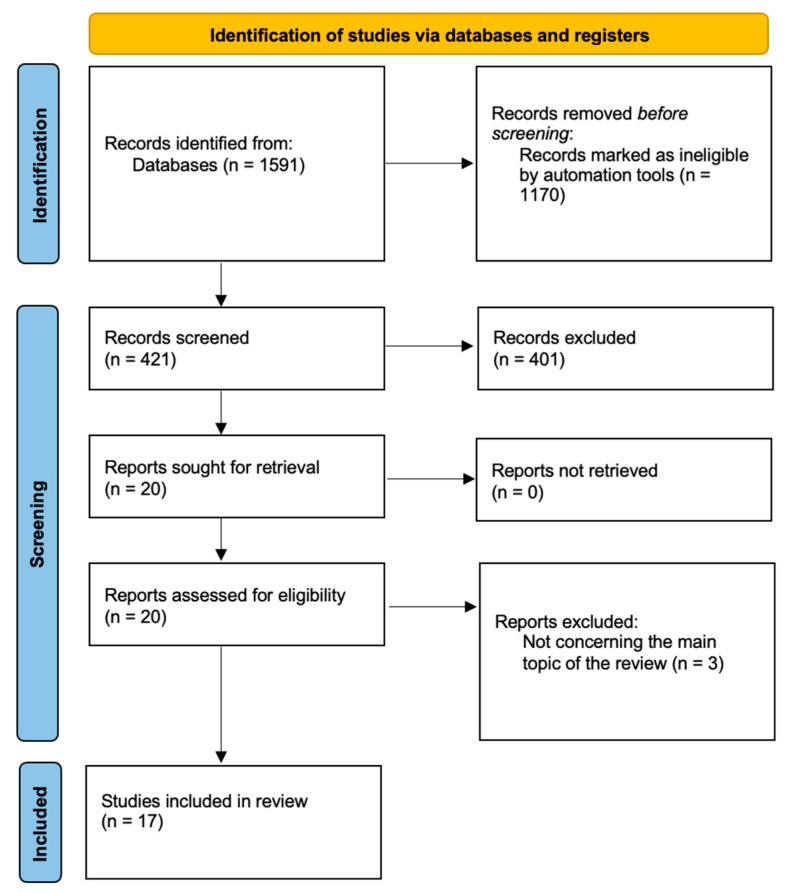
Study selection for long-term maternal consequences.

**Figure 3:**
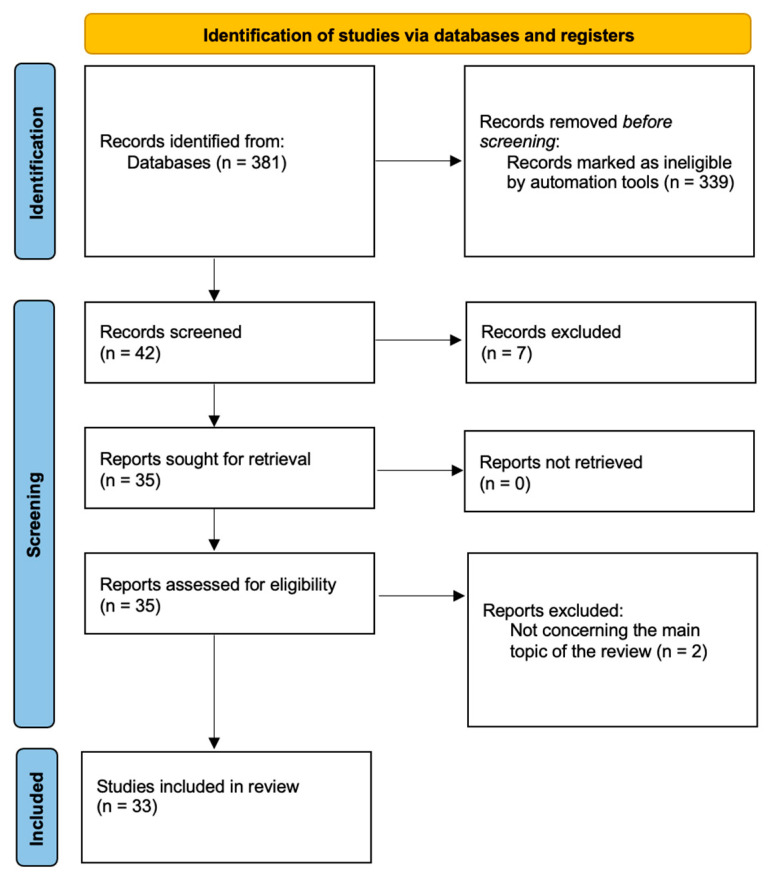
Study selection for fetal programming for metabolic diseases in children born to mothers with a history of EGWG.

**Figure 4:**
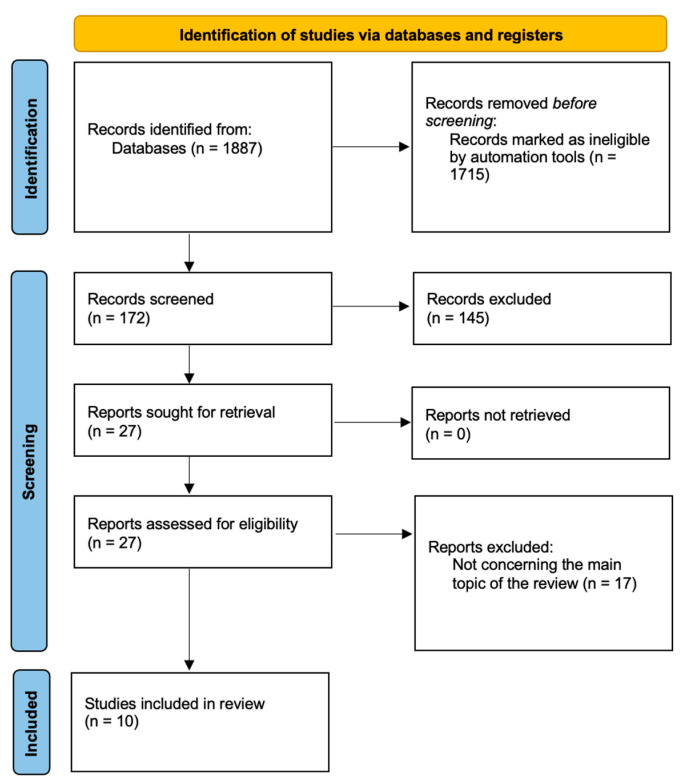
Study selection for comparison between the effects of pre-pregnancy overweight or obesity and EGWG.

Primarily, we intended to follow PRISMA guidelines for our review; however, we wanted to discuss EGWG-related aspects as comprehensively as possible and not limit ourselves to a certain topic regarding EGWG. We followed PRISMA guidelines in the subsections discussing dietary and exercise recommendations, maternal and fetal programming, as well as comparing the impact of pre-pregnancy overweight or obesity on EGWG development during pregnancy. We concluded that the subsection on EGWG pathophysiology did not quite fit into the specifics of PRISMA. As a result, we created four flow charts to demonstrate our search strategies (Figure 1, Figure 2, Figure 3 and Figure 4).

## 4. Pathophysiology and Risk Factors of EGWG

The pathophysiology of EGWG is a complex and not fully understood issue that is closely related to both the health and nutritional status before pregnancy and alterations arising in the pregnant mother’s body. As pregnancy advances, there is a concurrent increase in body weight, with the most significant rise typically occurring during the second trimester. During the initial trimester, weight gain primarily manifests as fat deposition, while later stages witness contributions from fetal growth and overall pregnancy development. Approximately half of the weight gain stems from the feto-placental component, a quarter from increased blood volume, extravascular volume, and breast tissue, while the remaining portion comprises the accumulation of cellular water, protein, and fat [6]. EGWG is an increasingly observed phenomenon and is estimated to affect up to 43% of pregnant women. Pre-pregnancy BMI > 25 is one of the most significant risk factors of EGWG, and the BMI before conception is the most important predictor of GWG in general [7,8,9,10,11]. According to Restall et al. [9], women who were overweight and obese at 14–16 weeks of gestation were, respectively, 3 and 2.5 times more likely to gain more gestational weight than recommended. Pre-pregnancy obesity, resulting from a high-calorie diet or genetic predisposition, alters lipid/energy metabolism during pregnancy [11]. During pregnancy, maternal weight influences levels of insulin, leptin, triglycerides (TGs), and c-reactive protein (CRP) [12].

Studies indicated that EGWG, maternal obesity, and GDM correlate with low-grade chronic inflammation, leading to macrophage infiltration and the release of adipokines. This is attributed to a disbalance between pro- and anti-inflammatory responses, which is also observed in pregnancy due to placental production of the tumor necrosis factor-α (TNF-α). Elevated interleukin 6 (IL-6) levels, observed in obese pregnant women, result in placental fatty acid uptake up-regulation that contributes to excessive fat deposition [13]. Abnormal adipose tissue accumulation is associated with the dysregulation of adipokines and an altered prooxidant–antioxidant system [14]. Moreover, EGWG, as well as maternal obesity, have dysfunction of feto-placental endothelium in common and, in particular, nitric oxide and endothelin-1 imbalance [13].

Leptin is a protein encoded by the obese (ob) gene, mainly produced in white adipose tissue, acting as the satiety signal [15]. It regulates food intake, thermogenesis, fatty acid oxidation, energy expenditure, glucose and lipids homeostasis, and body weight [16,17]. Hormones, including progesterone and estradiol, can influence the leptin synthesis. In pregnancy, leptin is also synthesized in the placenta and regulates placental nutrient transport and angiogenesis, trophoblast mitogenesis, and immunomodulation [18,19]. An increase in circulating free leptin concentrations correlates with BMI and fat mass and is commonly observed in obesity. Leptin levels throughout pregnancy rise, peaking in the second trimester, remaining at the plateau for the third trimester, and declining postpartum [19].

Behind this phenomenon is increasing body fat and the additional production of leptin by the placenta [15,20]. Increased leptin concentration, however, does not decrease energy supply, as a positive energy balance is necessary for fetal development, maternal health, and subsequent breastfeeding during pregnancy. Therefore, the condition is associated with temporary resistance to leptin in pregnancy, a physiological adaptation that acts as a compensatory mechanism, and might be related to the development of EGWG [18,21]. Leptin resistance is characterized by reduced brain sensitivity to leptin or general response failure, causing leptin’s failure to inhibit appetite or energy expenditure. It results in reduced satiety, increased food intake, and increased body mass, leading to obesity [15,17]. Franco-Sena et al. [22] reported that leptin levels correlate with weight gain in normal-weight and overweight pregnant women but not in obese women. Walsh et al. [23] reported higher leptin levels in healthy pregnant women with EGWG than women with adequate GWG, regardless of their BMI. Interestingly, a significant increase in its concentrations can also be observed in patients with preeclampsia [24]. Moreover, adiponectin levels in women with EGWG are lower compared to normal-weight patients, while hypoadiponectinemia is considered a GDM predictive marker [12,13,14].

Glucose–insulin metabolism, directly affecting body weight changes and composition, is modified during pregnancy by numerous proteins, hormones, and cytokines [13]. Hormones released by the placenta, such as human placenta lactogen (hPL), placental growth hormone (PGH), and placental corticotropin-releasing hormone (CRH), affect tissue insulin sensitivity and the formation of insulin resistance but also regulate gluconeogenesis and lipolysis. Their action may contribute to the occurrence of insulin resistance in pregnancy [13]. Women affected by obesity have higher concentrations of glucose transporter 1 (GLUT1) receptors and sodium-coupled neutral amino acid transporters (SNAT), and their maternal adipose tissue is involved in increasing insulin resistance [11]. Perichart-Perera et al. [12] observed elevated insulin levels in pregnant patients with EGWG. Elevated insulin levels, together with increased estrogens and progesterone levels, throughout the “anabolic” first and second trimesters induce lipid deposition that facilitates the cellular uptake of TGs [13]. Cade et al. [25] found a positive correlation between EGWG and a greater decrease in insulin sensitivity between 15 and 35 weeks. Various reports revealed that pregnant women with type 1 diabetes mellitus (T1DM) gain more weight than healthy patients [26,27,28]. Possible reasons include increased insulin doses, recurrent hypoglycemia, reduced physical activity, or erratic carbohydrate counting. More intensive insulin therapy, introduced during pregnancy, is also associated with greater weight gain compared to the standard dosage [27]. Of the 633 women with T1DM and type 2 diabetes mellitus (T2DM) studied by Xie et al. [29], 42.5% had EGWG. Among the predictors of EGWG, the authors pointed to height, pre-pregnancy BMI, multiple pregnancies, and pump use and insulin dose in the third trimester.

Psychosocial aspects should also be considered when addressing the excessive weight gain risk factors during pregnancy. Depression, body image dissatisfaction, and a lack of social support are strongly linked to EGWG occurrence [30]. Worse socio-economic status, living in low- or middle-income countries, limited or lacking access to healthcare, and migrant status within the past 5 years are associated with an increased risk of EGWG as well [9,31]. Other important risk factors are maternal eating habits, such as a lipid-based diet, increased food intake (“eating for two”), or a more sedentary lifestyle in pregnancy [32,33].

It is worth noting that distinguishing between GWG and maternal edema is essential for the proper diagnosis; however, the presence and scale of edema are not addressed in many studies. A simple measurement of weight on a body scale does not determine whether the weight gain is an effect of increased sodium and water retention, which are common in pregnancy, or increased body fat [34].

**Figure 5:**
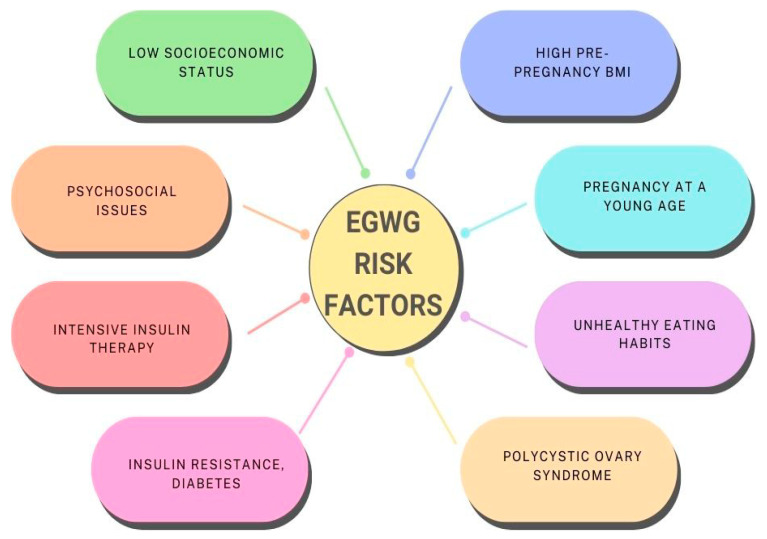
Common EGWG risk factors.

Common risk factors for EGWG are presented in Figure 5.

## 5. EGWG Prevention—Dietary and Training Recommendations

### 5.1. Physical Activity

There are many studies presenting the beneficial effects of physical activity during pregnancy. Exercise benefits not only the woman by helping to maintain adequate GWG but also the fetus. However, it is crucial to appropriately select activities due to the anatomical and physical changes occurring during gestation. Ha et al. [35] showed that physically active pregnant women gained about 0.5 kg less than less active women. Women who spent more time in a sitting position gained approximately 0.6 kg more than women who led an active lifestyle. A probable mechanism responsible for the benefits of exercise in pregnant women is a reduction in leptin levels.

In 2021, the results of a prospective study in Taiwan were published, which investigated the association between reduction in physical activity during pregnancy with EGWG occurrence. A total of 747 women in the second trimester of gestation were qualified for the study, of whom as many as 41.2% had EGWG. Researchers presented that the cause of EGWG was a reduction in their physical activity levels by >4000 Metabolic Equivalents of Task min/week (MET). Therefore, in low-risk pregnant women who were already active before pregnancy, it is important to maintain their level of physical activity also during pregnancy to avoid EGWG [36]. Another meta-analysis shows that exercising three times a week for 30–45 min may bring some benefits and reduce GWG in pregnant women [37].

The results of the FitMum research seem to be interesting, as they mostly contradict the results of previous studies. The authors conducted a randomized controlled trial designed to determine the effect of supervised exercise training (EXE) three times per week with motivational counseling on physical activity (MOT) compared to standard obstetric care. The final GWG results at 40 weeks + 0 days were 14.9 kg for CON, 15.7 kg for EXE, and 15.0 kg for MOT. The study did not show that either supervised exercise training or motivational counseling influenced GWG in pregnant women. In the EXE and MOT groups, moderate-to-intense physical activity was less than 1 h per week, which may be the actual reason for their lack of influence on GWG. It should also be taken into account that the study was conducted during the COVID-19 pandemic; however, the authors denied its impact on the amount of physical activity in the respondents. It is also worth noting that among the Danish population, the incidence of overweight or obesity is lower compared to women from other countries [38]. The prevalence of overweight among adult females in Denmark is 47.3%, compared to the United States of America, which is 63.2%, and in the United Kingdom, this number is 58.9% [39].

The American College of Obstetrics and Gynecology (ACOG) recommended an exercise program of at least 20–30 min a day on most days of the week in 2015 [40]. There are specific ACOG guidelines for obese pregnant women. When exercising, it is recommended to start training with short periods of low-intensity exercise and gradually increase the duration and intensity of exercise [40]. Aerobic and strength exercises, as well as walking, cycling, and swimming, are safe and might be beneficial [41]. Moreover, 2017 guidelines add resistance and stretching exercises, dancing, and hydrotherapy to that list [42]. While yoga combined with other forms of physical activity may help prevent weight gain in non-pregnant people, there is no evidence of the same benefit in pregnant women. A review by Green et al. [43] showed that prenatal yoga is not effective in preventing EGWG. To exclude possible contraindications to exercising, it is crucial to collect a clinical interview. In case of certain anatomical or physiological abnormalities of the fetus, exercises should be modified; however, it should not be a routine regimen in an obstetrician-gynecologist’s office but only used in high-risk pregnancies [40]. Skiing, surfing, or diving are not recommended due to the risk of falling or getting injured. It should also be consulted with a doctor, which exercises may cause reduced venous return or hypotension [44,45]. There are concerns that too much physical activity during pregnancy may contribute to complications in newborns, including small for gestational age (SGA). Clapp et al. [46] reported that exercise may cause reduced delivery of oxygen and other essential nutrients to the placenta, which may result in poorer fetal development. For this reason, it is necessary to remain vigilant and routinely monitor pregnant women who engage in intense physical activity.

### 5.2. Diet and Nutrition

The literature indicates a huge impact of the mother’s diet on development and the likelihood of possible pregnancy complications [47,48]. Lin et al. [49] showed that the fetus is extremely susceptible to the mother’s improper nutrition. Therefore, it is important that a pregnant woman adapts to the nutrition guidelines and provides the appropriate amount of calories, which can prevent EGWG. In pregnancy, the maternal basal metabolic rate increases by 10–20%, and caloric requirements increase by 85 kcal/day, 285 kcal/day, and 475 kcal/day in the first, second, and third trimesters, respectively [6].

The intake of folic acid is important and should be 400 μg per day in pregnant women. Supplementation of 150 μg iodine per day is also recommended. There is no need to supplement calcium during normal pregnancy, but it should be advised if women is at risk of hypertension. Iron supplementation should be based on haemoglobin concentration [50]. To our knowledge, there are currently no studies describing the relationship between vitamin D concentration in pregnant women and the development of EGWG. However, Shao et al. [51] showed that vitamin D deficiency in overweight and obese women before pregnancy may significantly affect the risk of developing GDM. Moreover, an analysis by Rodrigues et al. [52] indicated the relationship between vitamin D deficiency in a pregnant woman and higher insulin concentration and, therefore, an increased risk of insulin resistance in the third trimester of pregnancy. Considering these results, women should be advised to start vitamin D supplementation during the preconception period. This may not only allow the acquisition of better metabolic results but also the possibility of reducing the risk of EGWG.

Proper hydration during pregnancy is also crucial. The statement of Polish Gynecologic Experts indicates that the amount of water consumed during pregnancy should increase by approximately 300 mL/day [53]. In turn, in the second and third trimesters, it is necessary to drink about 3 L of water a day. Pregnant women do not have to limit their salt intake, but the use of iodized salt [54].

When it comes to the daily diet, the inclusion of ingredients such as fruit, vegetables, whole grain products, nuts, legumes, and fish is recommended [55]. Considering that these are nutritious products that make one feel full for longer, it can prevent a pregnant woman from snacking on other meals or sweets and, thus, prevent EGWG. Simple sugars and fast food in a diet should also be avoided.

The ProcriAr Cohort Study described the impact of nutrition on 385 pregnant women in Brazil. The study identified three dietary patterns: “vegetables and fruits”, “Western”, and “Brazilian Traditional”. The Western model was rich in processed meats, sweets, carbonated drinks, and snacks. In turn, “Brazilian style” was associated with a greater consumption of rice or beans. The most beneficial effect on EGWG was demonstrated by the “Brazilian style” and age > 35 years [56].

Another study aimed to determine the impact of the Mediterranean diet (MD) during pregnancy on GWG and postpartum weight retention (PPWR) in 243 patients. The results indicated a lower likelihood of EGWG with the use of MD and medical consultations. Vegetables and olive oil consumption were most effective in EGWG prevention, while legumes and whole grain products did not contribute to the study outcomes [57]. Dietary patterns are essential for pregnant women to maintain adequate weight gain. The available recommendations should be disseminated in the obstetrician’s office, and patients should be made aware of the risks resulting from improper nutrition and, thus, EGWG.

### 5.3. Remote Methods of GWG Control

Telehealth systems seem to be an interesting option to control GWG. In 2021, the results of a two-arm parallel randomized controlled trial determining the impact of using a smartphone application (the HealthyMoms app) on pregnant women were published. The intervention group, which included 152 women, received the HealthyMoms application for 6 months, while the control group, consisting of 153 women, received standard obstetric care. The study results showed no significant effect on GWG overall; however, women with pre-pregnancy overweight or obesity gained less weight in the intervention group than controls [58].

The GLOW study also aimed to determine the impact of a telehealth lifestyle intervention on GWG in women. The rate of GWG in women in the study group was, on average, 0.26 kg per week, while in controls, it was 0.32 kg per week. Patients observed not only a reduction in total caloric intake or the percentage of calories from saturated fat but also a reduction in a sedentary lifestyle. Moreover, improvements in insulin resistance markers, leptin levels, and cord blood C-peptide levels were observed in women in the intervention group [59].

Results of both studies [58,59] showed that telehealth interventions might be beneficial in the prevention of EGWG in some women. Interventions using electronic methods may have advantages due to easy accessibility and cost-effectiveness. Although electronic systems had an impact on, for example, dietary results in patients, there was no effect on Moderate-to-Vigorous Physical Activity (MVPA). Therefore, it seems that electronic weight control methods will play a greater role in building eating habits in patients than in building habits related to physical activity. This intervention method may be beneficial due to its ease and wide availability, and it might be considered as an addition to help patients plan and monitor weight gain or the amount of daily physical activity.

Dietary and training recommendations in EGWG prevention are summarized in Table 2.

## 6. Long-Term Maternal Consequences

GWG includes maternal fat accumulation, weight of amniotic fluid, placenta, and growing fetus. It is unquestionably necessary for the proper course of pregnancy, but if it is excessive, it can be associated with negative consequences for mothers’ health [60]. In just 15 years, GWG rates have increased. According to a retrospective cohort study in Germany that included 583,633 women with singleton pregnancies, the rate of women with EGWG rose from 64.54 to 68.55% between 2000 and 2015. This had a significant impact on increasing the incidence of cesarean sections and short-term obstetric outcomes [61]. It is well known that pregnancy and the postpartum periods are highly correlated. If metabolic changes during pregnancy are not controlled, they can also have adverse consequences for postpartum women and raise the risk of developing chronic disease in the future. Moreover, every preventive action before and during pregnancy can have a positive long-term impact on the health of the mother and infant [62].

Women with EGWG retain more weight postpartum compared to women with adequate GWG, according to the Institute Of Medicine’s (IOM) recommendations [63]. According to research, only 11% of overweight and obese pregnant women return to their preconception weight within 5 years postpartum [64]. A comprehensive meta-analysis involving more than 69,000 women indicated the excess weight gained in pregnancy is still retained even 20 years later [63]. In a multiethnic study of 1181 women who reported 2693 births overall, it was found that nearly half (47.6%) of the women who had EGWG had higher (obese) BMIs at midlife (age 42–53) than the 22.9% of women who had never experienced EGWG [65]. Furthermore, another analysis found that each pregnancy with EGWG in a woman’s life increased the odds of obesity at midlife to 64%, so in the case of several pregnancies in the reproductive history, the effect can be cumulative. This suggests that the total number of pregnancies with EGWG can have more influence on maternal weight than the other with adequate GWG across pregnancies [65]. According to The Women’s and Infants’ Study of Healthy Hearts (WISH) [66], women with EGWG history are at a three-fold higher risk of developing abdominal obesity within 8 years after childbirth compared to women with GWG.

It is well known that the development of visceral fat increases the risk for negative pregnancy-related outcomes, including GDM [67]. Harper et al. [68] showed that 368 of the 635 women with GDM diagnoses gained more weight than recommended by the IOM. As a result, the incidence of pregnancy-related hypertension increased from 36 to 83% for every pound gained in weight per week following a diagnosis of GDM. Higher abdominal adiposity postpartum plays an integral part in the development of impaired glucose tolerance, insulin resistance, T2DM, and various co-morbidities such as cardiovascular disease and metabolic syndrome as the woman ages [62,69].

Metabolic syndrome is characterized by abdominal obesity, increased blood pressure, abnormal circulating lipid parameters and serum plasma glucose, and insulin resistance [70]. While there is a lack of conclusive research on the effects of EGWG on TGs, a recent study conducted on overweight pregnant women revealed that mothers who gained excessive weight also had higher baseline TGs (81.7 ± 47.2 vs. 69.7 ± 40.3 mg/dL) than women of normal weight [71]. The role of metabolic activity of subcutaneous tissue and increased postpartum weight retention in women with EGWG can be explained by the results of an Australian study. Women with EGWG had a 47% higher chance of presenting with T2DM within 21 years of giving birth compared to controls in a birth cohort of 7223 mothers [72].

Additionally, more and more studies show the association between EGWG and the risk of developing postpartum depression (PPD) [73]. There is some evidence of EGWG being a significant predictor of postpartum weight retention, which might contribute to the development of PPD. It is also worth noting that EGWG may negatively affect the body image of pregnant women, which could reduce their self-esteem, enhance the possibility of body dissatisfaction, and exacerbate mood disorders [74,75].

Minschart et al. [76] found out that women with high postpartum weight retention breastfed less frequently, had a more impaired postpartum metabolic profile, higher depression rates and anxiety levels, and lower quality of life. Other researchers examined the relationships between postpartum psychological dimensions; symptoms of anhedonia, anxiety, and depression; and adequate, inadequate, and excessive GWG at the time of pregnancy using the Edinburgh Postnatal Depression Scale (EPDS0), a validated screening tool. The sample included 1268 mothers of healthy infants delivered at term, 557 (43.9%) with adequate GWG, 388 (30.6%) with inadequate GWG, and 323 (25.5%) with EGWG. Results showed that women with EGWG history are at risk of developing anxiety and anhedonia, while women who experienced inadequate GWG have a higher risk of developing early postpartum depression [77].

## 7. Fetal Programming for Metabolic Diseases in Children Born to Mothers with a History of EGWG

EGWG is not only associated with the risk of maternal complications but also poses a threat to the offspring [8,78]. Intrauterine life was proven to be one of the most crucial stages of development that may significantly affect patients’ future health [79]. Non-communicable chronic diseases such as obesity that were formerly linked to genetics and lifestyle were shown to have early life origins [80]. The adverse intrauterine environment exerts influence on the fetus, undergoing a critical developmental period when rapid cell division occurs, leading to rather irreversible adaptations in homeostatic systems, organs, and tissues. This is referred to as “fetal” or “metabolic programming”, and the mechanisms behind it are supposedly connected with hormonal factors, the gut microbiome, inflammation, and epigenetic modifications [80,81].

EGWG seems to correlate with cord blood hormone levels, which may be one of the determinants of a child’s later health [82]. Various cohort studies showed that GWG above recommendations increases the probability of cord hyperleptinemia [83,84,85]. According to one study, not only leptin but also ghrelin cord blood concentrations are increased in infants born to mothers with EGWG, but that only applies to male neonates [86]. A prospective study including 978 mother–infant pairs conducted by Rifas-Shiman et al. [87] revealed that higher GWG in the first trimester was linked to lower adiponectin, higher insulin, and c-peptide, whereas higher GWG in the second trimester was related to higher levels of leptin, insulin-like growth factor-1 (IGF-1), IGF-2, and insulin-like growth factor-binding protein 3 (IGFBP-3). This may implicate that early pregnancy weight gain more markedly affects offspring’s glucose homeostasis, while mid–late pregnancy weight gain contributes to alterations in hormonal determinants of growth and adiposity.

One of the mechanisms known to modify fetal growth is the transfer of nutrition and oxygen-rich umbilical venous blood to the fetal liver. A higher umbilical flow to the liver was proven to correlate positively with newborn adiposity [88]. A longitudinal observational study including 49 women found that EGWG increases umbilical flow, umbilical flow to the liver, and left portal vein velocity, which is predisposed to the higher birthweight of neonates. However, the participants were affected with pre-gestational diabetes mellitus, and this relationship was not as pronounced in the reference population [89].

Nevertheless, the link between EGWG and neonatal adverse outcomes, including fetal macrosomia and large-for-gestational-age (LGA) neonates, seems unquestionable. According to a meta-analysis of 23 cohort studies, summarizing data from 1,309,136 pregnancies from the United States, Europe, and Asia, EGWG increases the risk of LGA, defined as birth weight greater than the 90th percentile for gestational age, and macrosomia, described as birth weight greater than 4000 or 4500 g. These correlations were found to be stronger in women with lower pre-pregnancy BMIs [90]. Another meta-analysis of 39 cohorts covered data from Europe, North America, and Oceania to evaluate the separate and combined impact of maternal BMI before pregnancy and GWG on adverse pregnancy outcomes. Aside from causing threats to the mother (gestational hypertension, preeclampsia, GDM), EGWG was proven to predispose to a higher risk of LGA at birth. A total of 31.6% of cases of LGA infants examined were attributed to their mothers’ weight gain exceeding recommendations. Moreover, pregnancy complications were most likely to occur in obese women who gained excess weight [91].

A Chinese population-based prospective cohort study was designed to analyze the impact of EGWG on pregnancy short-term outcomes, with the distinction between the stage of pregnancy (early: ≤17 weeks and late >17 weeks of gestation) and fetal sex. Data on 2630 women indicated that EGWG in early pregnancy more severely affects maternal health, while later weight gain impacts a neonate, increasing the risk of a cesarean section, and LGA, regardless of fetal sex and macrosomia, especially in male fetuses [92]. Another research work revealed that a higher prevalence of macrosomia and LGA in neonates born to mothers with GDM who gained excess weight during pregnancy is additionally increased by concurrent overweight or obesity as well as abnormal results from an oral glucose tolerance test (OGTT) [93].

Formerly, EGWG was shown to affect not only offspring’s body weight but also body composition. In 2014, Badon et al. [94] used neonatal anthropometric measurements, including the sum of flank, subscapular, and triceps skin fold thicknesses; birthweight; and body fat percentage, to examine neonatal adiposity within 72 h of delivery. Healthy and overweight women who gained more weight than is recommended had an increased probability of delivering infants with the sum of skin folds and percentage body fat > 90th percentile. Similar results were obtained by Henriksson et al. [95], who performed air-displacement plethysmography in 312 one-week-old neonates to evaluate their body composition and refer it to the GWG of their mothers. For women who were of normal weight before pregnancy, EGWG was linked to increased infant fat mass. According to a calculation by Carlsen et al. [96], who assessed the neonates’ body composition using dual-energy X-ray absorptiometry, there is an 11 g rise in newborn fat mass for every kilogram of the mother’s GWG.

Due to the scientific research available, increased birth weight can have long-term consequences for metabolic health. It was proven that infants born LGA have a higher chance of growing up to be overweight or obese, as well as developing metabolic syndrome in adulthood [97]. So far, numerous studies have shown a connection between EGWG, usually in combination with an abnormal pre-pregnancy body weight, and obesity prevalence in offspring [98,99,100]. A large cohort study by Bider-Canfield et al. [101], including 15,710 mother–infant pairs, revealed that although the strongest risk factor for childhood overweight at the age of 2 years is maternal obesity or overweight, EGWG may also contribute to the offspring’s abnormal weight, which is predominantly mediated through birth weight and gestational age at delivery. A meta-analysis of 12 studies was performed to verify the relationship between EGWG and the risk of obesity in offspring within three life stages: <5 years, 5 to <18 years, and above 18 years. Overall, findings showed that children of mothers who gained weight above guidelines are 40% more likely to develop obesity compared with the reference group of adequate GWG mothers. The elevated incidence of obesity concerned each age group; however, the association’s strength decreased slightly with increasing time after birth [102].

Inconsistently, a subsequent, larger meta-analysis of individual participant data from 37 pregnancy and birth cohort studies conducted in Europe, North America, and Australia, involving 162,129 mothers and their infants divided into three groups, 2.0–5.0 years, 5.0–10.0 years, and 10.0–18.0 years, demonstrated that the discussed association is the most significant in late childhood. The authors attributed these results to intra-uterine programming, which manifests as children become older, or the greater impact of lifestyle choices later in life [103].

**Figure 6:**
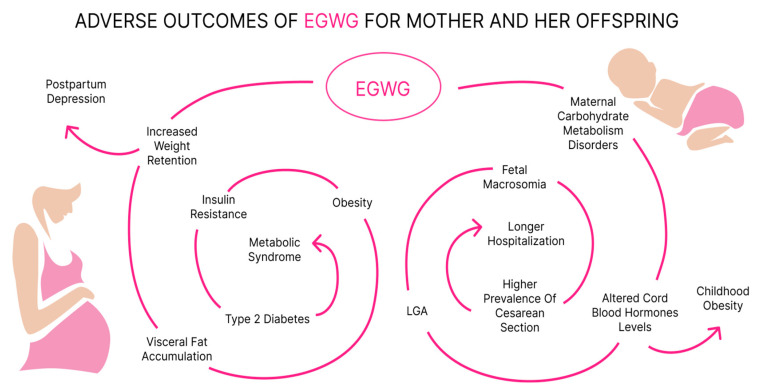
Common EGWG complications for mother and her offspring.

Common adverse outcomes of EGWG are presented in Figure 6.

## 8. Comparison between the Effects of Pre-Pregnancy Overweight or Obesity and EGWG

There is no doubt that women with pre-pregnancy overweight and obesity are at risk of adverse pregnancy outcomes. It is also well known that they are predisposed to EGWG and then postpartum weight retention, as these disorders are closely related. However, limited studies look for an independent effect of EGWG on maternal health and fetus development.

Gestational hypertension and preeclampsia are significantly correlated with pre-pregnancy obesity and EGWG, as well as chronic hypertension, a history of preeclampsia in previous pregnancies, older maternal age, and a family history of preeclampsia [104,105]. Preeclampsia, characterized by hypertension and proteinuria, typically manifests after 20 weeks of gestation. Its onset poses significant risks to both maternal and fetal health, potentially endangering their well-being and survival. Shao et al. [105] found that pre-pregnancy overweight and EGWG were independent risk factors for preeclampsia, with a potential synergistic effect from their co-occurrence. Swank et al. [106] showed that excessive BMI change during pregnancy increased the risk of developing gestational hypertension and preeclampsia, which also applied to women with normal pre-pregnancy BMI. A study by Lewandowska et al. [104] revealed that pre-pregnancy BMI was the most significant clinical parameter predictive of the development of gestational hypertension (including BMI ≥ 25 kg/m^2^), as well as preeclampsia (including BMI ≥ 30 kg/m^2^). However, GWG was also presented as a significant indicator associated with the development of gestational hypertension.

Obesity can increase the risk of venous thromboembolism (VTE) due to sustained chronic inflammation and increased plasma levels of coagulation factors such as fibrinogen and factor VIII. It can also elevate the risk of postpartum VTE by two- to three-fold. Blondon et al. [107] compared the effects of pre-pregnancy BMI and delivery BMI on the occurrence of VTE. They showed that both BMIs independently contribute to the risk of postpartum VTE, with a more pronounced correlation observed with pre-pregnancy BMI. The authors believe that a combination of both parameters might be beneficial in estimating the risk of postpartum VTE and qualifying patients for thromboprophylaxis.

Maternal obesity can also cause breastfeeding complications [108,109]. The pathogenesis of this phenomenon is still not well understood; however, it is believed that fat mass can interfere with prolactin and oxytocin levels, which can lead to a delay in the onset of copious milk secretion, known as lactogenesis II, compared to women of normal weight. Delaying this process contributes to a reduction in total breastfeeding duration. Any breastfeeding (ABF) is defined as a combination of breastfeeding and infant formula or solid food, while exclusive breastfeeding (EBF) does not include additional nutrition. Tao et al. [108] revealed that pre-pregnancy obesity had a negative effect on breastfeeding in Chinese women by increasing the risk of delayed lactogenesis II and earlier ABF termination. In contrast, GWG alone did not affect ABF or EBF. Castillo et al. [109] compared the association of pre-pregnancy BMI and GWG on patterns and the duration of breastfeeding at 3 months old. They noted that children of obese mothers were more likely to be weaned compared to normal-weight women, and pre-pregnancy overweight and obesity were negatively correlated with ABF and EBF. However, they did not show any association of breastfeeding with GWG.

Obesity in children increases the risk of obesity in adulthood and its associated health consequences. Fetal macrosomia is believed to be one of many risk factors causing childhood obesity. Swank et al. [110] showed that an excessive BMI increase during pregnancy results in a higher likelihood of macrosomia regardless of pre-pregnancy BMI, while the co-occurrence of both disorders can have a synergistic effect. Gujski et al. [111] compared the fetal birth weight (FW)/placental weight (PW) ratio in diet-controlled GDM patients with pre-pregnancy obesity or EGWG to healthy women. They showed that women with EGWG had the highest likelihood of cesarean section of all groups because of fetal weight and prolonged hospitalization due to newborn condition. Research by Hunt et al. [112] presented that maternal pre-pregnancy BMI and GWG were factors independently and positively correlated with the occurrence of overweight and obesity in children.

Moreover, there is some evidence of the effects of pre-pregnancy overweight and obesity, as well as EGWG, on the neurodevelopment of offspring. Pre-pregnancy obesity results in poorer cognitive function and intelligence quotient (IQ) development in offspring, while EGWG may be an independent and significant risk factor for poorer academic performance in children. Hao et al. [113] showed that the offspring of mothers with EGWG are at risk of lower cognitive function, which could potentially be related to the occurrence of chronic inflammation during pregnancy due to metabolic dysfunction.

## 9. Discussion

Given the increasing prevalence of EGWG and its possible long-term impact on maternal and offspring health and well-being, newer and more effective strategies are needed to prevent its consequences. There are many studies demonstrating that metabolic programming for obesity, T2DM, and metabolic syndrome is associated with the occurrence of excessive weight gain during pregnancy [62,66,69,72,112]. This poses a significant problem, given that this programming affects not only the mother but also the developing fetus.

It seems that a woman’s incorrect nutrition will have a greater impact on the development of EGWG than an insufficient level of physical activity. Moreover, many women are still not aware of the risks of EGWG for both themselves and their babies, which is why it is necessary to improve the education and counseling of pregnant women by obstetricians. Recommendations regarding nutrition and the physical activity of future mothers should be introduced already in the antenatal period and continued postpartum, which could prevent complications in subsequent pregnancies. It is also important to take care of psychological aspects, such as stress and social support, as EGWG poses a risk for postpartum depression, which, in turn, can contribute to postpartum weight retention and further metabolic complications [76,77].

Special attention should be given to patients who are more prone to experience EGWG. In women with polycystic ovary syndrome (PCOS), EGWG is more prevalent and might relate to insulin resistance, mood, and eating disorders but also other neuro-hormonal gut–brain interactions [10,114]. There is some proof that metformin decreases serum leptin levels in non-pregnant PCOS women. Molin et al. [21] found out that normal-weight PCOS patients treated with metformin had a lower risk for EGWG due to its potential to improve leptin sensitivity in PCOS pregnant women. Therefore, the use of metformin during pregnancy may counteract leptin resistance and prevent EGWG in patients with PCOS.

While we attempted to conduct a comprehensive literature review of recent reports related to EGWG, we believe that our study has some limitations. First, the publications we selected include patients from certain demographics or socioeconomic backgrounds, which could affect the representativeness of the findings. Studies on EGWG focused primarily on short-term effects on pregnancy and the postpartum period, while there are insufficient data to present long-term consequences; therefore, we cannot make clear conclusions regarding long-term maternal and fetal programming. Also, self-reported data in some studies regarding dietary strategies to prevent EGWG posed a challenge, as it is not possible to verify with certainty whether all patients followed the recommendations.

Future studies should focus on forming as ethnically and environmentally diverse groups as possible, as well as include patients with all BMI categories, as this could provide a better understanding of which patients will benefit most from a given form of physical activity. A good solution is the increasingly frequent use of electronic forms of weight gain control, which can have a positive impact on motivation to exercise. By pursuing these future directions, researchers can contribute to advancing knowledge and improving outcomes related to gestational weight gain prevention, ultimately promoting the health and well-being of mothers and their offspring.

## 10. Conclusions

Our study highlights the importance of addressing EGWG to promote maternal and offspring health. We demonstrated the significance of interventions targeting nutrition, physical activity, and psychosocial factors in mitigating the risks associated with EGWG. Education and counseling remain vital components of prenatal care, necessitating ongoing collaboration between healthcare providers and researchers. By embracing interdisciplinary approaches and translating evidence into practice, we can advance efforts to prevent EGWG and improve outcomes for mothers and their offspring.

## Figures and Tables

**Table 1:** Institute of Medicine (US) and National Research Council (US) Committee to Reexamine IOM Pregnancy Weight Guidelines: Weight gain during pregnancy: reexamining the guidelines. Washington DC: National Academies Press, 2009, modified.

Recommended Weight Gain during Pregnancy		
Pre-pregnancy BMI	Recommended total weight gain during pregnancy (from the beginning of pregnancy to delivery)	Recommended average weekly weight gain in the II and III trimesters of pregnancy
Normal body weight (BMI 18.5–24.9 kg/m^2^)	11.5–16.0 kg	0.42 (0.35–0.50) kg/week
Overweight (BMI 25.0–29.9 kg/m^2^)	7.0–11.5 kg	0.28 (0.23–0.33) kg/week
Obesity (BMI > 30.0 kg/m^2^)	5.0–9.0 kg	0.22 (0.17–0.27) kg/week

**Table 2:** Recommendations of lifestyle intervention preventing EGWG summarized.

Authors, Publication Years, References	Recommendations
Ha et al. (2020) Sun et al. (2021) Wang et al. (2019) ACOG Committee Opinion No. 650 (2015) [35,36,37,40]	Physically active pregnant women gain less weight than non-active ones, which can prevent EGWG. It is recommended to exercise at least 20–30 min a day on most days of the week.
Roland et al. (2023) [38]	Supervised exercise training 3 times a week and motivational counseling on physical activity are not superior to standard obstetric care.
Vargas-Terrones et al. (2019) Berghella et al. (2017) Green et al. (2022) [41,42,43]	Activities that are safe and beneficial include aerobic, strength, resistance, and stretching exercises. Walking, cycling, swimming, dancing, and hydrotherapy are recommended. Yoga exercise turned out to be ineffective in EGWG prevention.
Department of Health Clinical Practice Guidelines: Pregnancy Care (2020) [50]	Supplementation of 400–800 μg of folic acid, vitamin B1, and iodine is recommended. There is no need to supplement iron or calcium during healthy pregnancy.
Shao et al. (2020) Rodrigues et al. (2022) [51,52]	Vitamin D deficiency in pre-pregnancy overweight or obese women may significantly affect the risk of developing GDM. Its deficiency in third trimester might cause higher insulin concentration and development of insulin resistance.
Hanson et al. (2015) Marshall et al. (2022) Saldiva et al. (2022) Radwan et al. (2022) [54,55,56,57]	In the second and third trimester water consumption should be about 3 L a day. There is no need to limit salt intake; however, salt should be iodized. It is recommended to include fruits, vegetables, whole grain products, nuts, legumes, and fish in diet during pregnancy. The traditional Brazilian diet consisting of rice and beans, as well as consumption of vegetables and olive oil, are proven to be effective in preventing EGWG.
Sandborg et al. (2021) [58]	HealthyMom app usage for 6 months had no significant effect on EGWG; however, women with pre-pregnancy overweight or obesity gained less weight compared to controls.
Ferrara et al. (2020) [59]	Study group using telehealth lifestyle intervention had reduced total caloric intake and sedentary lifestyle. They also obtained better leptin and blood C-peptide levels compared to controls.

EGWG—excessive gestational weight gain, GDM—gestational diabetes mellitus.

## Data Availability

Not applicable.
